# Design of Hydrogel Silk-Based Microarrays and Molecular Beacons for Reagentless Point-of-Care Diagnostics

**DOI:** 10.3389/fbioe.2022.881679

**Published:** 2022-07-22

**Authors:** Alicia Sampieri, Ricardo Monroy-Contreras, Alexander Asanov, Luis Vaca

**Affiliations:** ^1^ Departamento de Biología Celular y del Desarrollo, Instituto de Fisiología Celular, UNAM, Ciudad Universitaria, Mexico, Mexico; ^2^ TIRF Labs, Cary, NC, United States

**Keywords:** molecular beacons, microarrays, silk, point-of-care diagnostics, influenza virus

## Abstract

We have developed a novel microarray system based on three technologies: 1) molecular beacons designed to interact with DNA targets at room temperature (25–27°C), 2) tridimensional silk-based microarrays containing the molecular beacons immersed in the silk hydrogel, and 3) shallow angle illumination, which uses separated optical pathways for excitation and emission. Unlike conventional microarrays that exhibit reduced signal-to-background ratio, require several stages of incubation, rinsing, and stringency control, and measure only end-point results, our microarray technology provides enhanced signal-to-background ratio (achieved by separating the optical pathways for excitation and emission, resulting in reduced stray light), performs analysis rapidly in one step without the need for labeling DNA targets, and measures the entire course of association kinetics between target DNA and the molecular beacons. To illustrate the benefits of our technology, we conducted microarray assays designed for the identification of influenza viruses. We show that in a single microarray slide, we can identify the virus subtype according to the molecular beacons designed for hemagglutinin (H1, H2, and H3) and neuraminidase (N1, N2). We also show the identification of human and swine influenza using sequence-specific molecular beacons. This microarray technology can be easily implemented for reagentless point-of-care diagnostics of several contagious diseases, including coronavirus variants responsible for the current pandemic.

## Introduction

Undoubtedly, the current gold standard for molecular diagnosis is the polymerase chain reaction (PCR) (Kim, 2001). PCR, both in its traditional form and in what is known as real-time PCR, is a method widely used worldwide by clinics and hospitals for molecular diagnosis and identification of pathogens, mutations of interest, and polymorphisms (Grau-Roma et al., 2011).

The power of this diagnostic methodology is based on the fact that it is an amplification reaction, which gives it enormous sensitivity (Tong et al., 2011). PCR can detect a single nucleic acid molecule (Valasek and Repa, 2005) (Erlich et al., 1991).

However, PCR also has some problems that hinder its widespread use as a molecular diagnostic method. Below are listed some of the most important limitations of this powerful method:

1) PCR requires a thermal cycler, an expensive and bulky device that must be operated by a specialized technician.
2) The amplification reaction requires programming of the thermal cycler with heating and cooling cycles too complex to be operated by a doctor, nurse, or medical personnel. This amplification cycle is not universal and must be adapted for each DNA segment to be amplified based on the alignment and association temperatures of the oligonucleotides (known as primers) used to amplify the DNA of interest.
3) The amplification reaction (due to the temperature cycles mentioned above) takes more than 1 h to produce the result.

The ideal point-of-care diagnostics system should be one that requires very little sample preparation and handling, is portable and easy to use by any patient (Goble and Rocafort, 2017) (Ferreira et al., 2018). Many systems have been proposed during the last few years, some are easy to use and portable but require complex manipulations and labeling of the sample (Gauglitz, 2014).

One of the molecular recognition events with the highest specificity is the binding of a nucleic acid to its complement; this process has aided the exploration of gene expression and many laboratory methodologies are based on it (Chidchob and Sleiman, 2018). The potential of this technique has not been exploited efficiently for molecular diagnostics (Goble and Rocafort, 2017). To determine if a probe is hybridizing, it is necessary to label the target, immobilize the target complementary sequences on a solid surface, remove the target excess that did not hybridize, and define the number of probes to be used in the assay.

MBs are single-stranded nucleic acid molecules that form a stem-loop structure known as a hairpin (Bonnet et al., 1999; (Tyagi and Kramer, 1996; Monroy-Contreras and Vaca, 2011). The loop portion acts as a probe complementary to a predetermined target sequence (Tyagi and Kramer, 1996). The stem is formed by the union of a series of complementary bases that are located in the loop sequence (Tyagi and Kramer, 1996). The 5′ end of this molecule is covalently coupled to a quencher and the 3′end to a fluorophore. Under resting conditions (without a target), the quencher is in close proximity to the fluorophore, preventing the emission of fluorescence by a phenomenon known as FRET (Foster Resonance Energy Transfer) (Tyagi and Kramer, 1996; Bonnet et al., 1999). Upon MB-target hybridization, the stem from the MB dissociates, allowing the separation between the fluorophore and the quencher, resulting in fluorescence emission (Bonnet et al., 1999).

MBs are extremely specific and can easily discriminate target sequences that differ by a single nucleotide substitution (Vaca, 2014), and are more specific than any conventional probe. Thermodynamic analyzes reveal that this increase in specificity is due to the stem-loop (hairpin) conformation of MBs (Bonnet et al., 1999).

MB can be synthesized with different types of fluorophores, which allows simultaneous detection of different target molecules in a multiplex reaction (Monroy-Contreras and Vaca, 2011).

Because MBs are primarily used in PCR reactions, the length and the GC content of the stem sequence are designed in such a way that at the annealing temperature of the PCR, the MB remains closed and therefore non-fluorescent. This is generally ensured by choosing a stem 5–7 base pairs long that contains a high GC content (typically between 75 and 100% GC content) (Bonnet et al., 1999).

Unfortunately, this MB design prevents its use in target identification assays conducted at room (or near room) temperature. This is one of the reasons why MBs have not been employed in point-of-care diagnostics (Asanov et al., 2012).

In the present study, we have explored the length and GC content of MBs to identify the best conditions for target identification in assays conducted at room temperature (25–27°C).

DNA microarray is a multiplex technology used in molecular biology and medicine to identify DNA fragments expressed in an organism or tissue or present in fluids (Plomin and Schalkwyk, 2007). Microarrays consist of an ordered series array of hundreds or thousands of microscopic spots of DNA oligonucleotides (known as probes), each containing a few picomoles of different probes printed on top of silica slides (Gershon, 2002).

The sample is labeled using fluorescent molecules, which are later used to identify sample association with the probe printed on the silica slide (Gershon, 2002). Typical microarray spots are 100 microns in diameter, allowing the printing of thousands of spots in high-density arrays. But microarrays are not very sensitive and sample amplification is often required prior to conducting the microarray study.

Silk is a natural fiber essentially made up of two proteins: fibroin and sericin (Sutherland et al., 2010). Several insects and arthropods naturally produce silk; among them, the most commonly used is the *Bombix mori* larva, also known as the silkworm (Vepari and Kaplan, 2007). This larva produces its own silken cocoon, where it metamorphoses into a butterfly. Silk is an extraordinary product due to its properties as a biomaterial, since it can produce a wide variety of products such as sponges, gels, fibers of various diameters, and three-dimensional (Helmer, 2010; Lee, 2010; Sutherland et al., 2010). For centuries, silk has been used to produce clothing, but recently it has gained great interest because many biomaterials can be produced from it. Silk is traditionally extracted from the cocoon of the silkworm through a process of boiling and chemical extraction (Numata and Kaplan, 2010).

Silk gels are of particular interest since they have almost the same refractive index (1.34) as that ofwater (1.33). Therefore, light passes through silk gels similarly to water without any quenching (Uddayasankar and Krull, 2013; Beaucage, 2001).

The technology presented in this study utilizes MBs specifically designed to interact with their target at room temperature. The MBs are embedded in 3D microarray silk hydrogel matrices, favoring efficient fluorescence transmission and providing a friendly environment for biomolecular interactions.

We show the identification of cDNA from influenza subtype viruses, its selectivity, and its sensitivity.

## Materials and Methods

### Molecular Beacons

Synthesized and purified molecular beacons and complimentary DNA oligomers were purchased from Integrated DNA Technologies (IDT, Coralville, IA, United States). All the beacons in the present study use as fluorophore 6-carboxyfluorescein (6-FAM) at the 5′end, and as quencher 4-((4-(dimethylamino)phenyl)azo)benzoic Acid (Dabcyl) at the 3′end.

### Designing Molecular Beacons Used for the Analysis of GC Content and Overlap of Complementary Sequences

To explore the role of the guanine (G) cytosine (C) content in the stem of MB in the kinetics of MB-target formation, we designed MBs with 3 different percentages of GC content (40,60, and 100%). The loop section of the MBs was the same in all of them. The MB used in these experiments was

$$Q-3^{\prime }-\mathrm{gagagAAACCAATAGATCGACATActctc-}5^{\prime }\mathrm{-F}\;\left(40\text{\%}\;\text{GC}\;\text{content}\right)$$

$$Q-3^{\prime }-\mathrm{aggagAAACCAATAGATCGACATActcct-}5^{\prime }\mathrm{-F}\;\left(60\text{\%}\;\text{GC}\;\text{content}\right)$$

$$Q-3^{\prime }-\mathrm{gggggAAACCAATAGATCGACATAccccc-}5^{\prime }\mathrm{-F}\;\left(100\text{\%}\;\text{GC}\;\text{content}\right)$$

In all caps is shown the sequence of the loop in the MB and in lowercase the sequence corresponding to the stem.

To explore the overlapping of the target in the loop and stem from the MB, the following target sequences were designed:


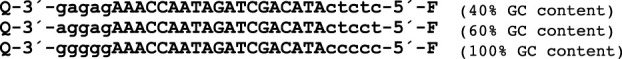


The sequences from the targets that are complementary to the loop from the MB are shown in all caps. Lowercase shows the sequences complementary to the stem region of the MB. The numbers to the right indicate the number of nucleotides overlapping with the stem from the MB, both unilateral (first 6 target sequences) and bilateral (five next target sequences).

### Designing Molecular Beacons for the Identification of Influenza Virus Subtypes

We follow a bioinformatics strategy to identify the best sequences in the different influenza genes to ensure high specificity when designing the MBs.

Influenza virus sequences were obtained from the Influenza Virus Resource: http://www.ncbi.nlm.nih.gov/genomes/FLU/FLU.html. All available sequences of the hemagglutinin HA (H1, H3) and neuraminidase NA (N1, N2) genes were downloaded from the aforementioned database and aligned using the command line version of the ClustalW program. The most conserved regions of each HA and NA subtype were selected using BioEdit software, from the previously generated alignments. Each of the conserved sequences was analyzed using the mfold server http://dinamelt.bioinfo.rpi.edu/quikfold.php; this analysis served as a filter to obtain those conserved sequences that contain a minimum secondary structure that does not interfere with the hairpin formation of the MBs. Some of these conserved sequences did not work to specifically detect each virus subtype since large regions are conserved in the gene. Due to this, it was necessary to carry out an “*in silico*” coverage analysis (Durai and Schulz, 2019), to ensure the specificity of the sequences for each of the influenza virus subtypes. The best sequences obtained after the analysis are shown in the table below:

**Table:** 

Subtype	Sequence	Length (nt)
H1	ATT​CGC​ATT​CTG​GGT​TTC​CTA​AGA​TCC​A	28
H3	CAT​TCC​CTC​CCA​ACC​ATT​TTC​TAT​GAA	27
H1s	TGA​AAT​GGG​AGG​CTG​GTG​TTT​ATA​GCA​C	28
N1	TCT​CTT​ATG​ACA​AAA​ACA​TCT​CC	23
N2	GAA​AAA​GGT​GCA​AAT​CCT​GTA​AT	23
N1s	TAC​TGT​ATA​TAG​CCC​ATC​CAC​TAA​CA	26

The “*in silico*” coverage analysis help define the sensitivity and specificity of each sequence (Durai and Schulz, 2019). The results obtained are illustrated in the table below:

**Table:** 

MB ID	Sensitivity %	True positives	Number of sequences analyzed	Specificity %
H1	96.17	1,279	1,330	99.83
H3	99.5	2,458	2,583	100
H1s	100	500	500	99.72
N1	98.06	1,417	1,445	100
N2	98.8	2,880	2,915	100
N1s	99.8	499	500	100

These sequences were used as the loop portion of the MBs, and a stem sequence was added to produce the final MBs sequences shown below (these MB sequences are protected by patent 344,352):

**Table:** 

MB ID	MB (5'--->3′) Sequence	Length (nt)
H1	CGT​AGCATT​CGC​ATT​CTG​GGT​TTC​CTA​AGA​TCC​AGC**T**ACG	40
H3	CGT​AGCCAT​TCC​CTC​CCA​ACC​ATT​TTC​TAT​GAAGC**T**ACG	39
H1s	CGT​AGCTGA​AAT​GGG​AGG​CTG​GTG​TTT​ATA​GCA​CGC**T**ACG	40
N1	CGT​AGCTCT​CTT​ATG​ACA​AAA​ACA​TCT​CCGC**T**ACG	35
N2	CGT​AGCGAA​AAA​GGT​GCA​AAT​CCT​GTA​ATGC**T**ACG	35
N1s	CGT​AGCTAC​TGT​ATA​TAG​CCC​ATC​CAC​TAA​CAGC**T**ACG	38

Underlined are the influenza genes’ complementary sequences corresponding to the loop regions of the MBs.

### Silk Preparation Hydrogels

There are different methods to generate silk hydrogels under controlled conditions. In the present study, we induced the hydrogel formation with methanol, as previously described (Numata et al., 2014; Numata and Kaplan, 2010)**.** Briefly, cocoons from the silkworm *Bombyx mori* were grounded and boiled for 30 min in a 20 mM Na_2_CO_3_ solution and then washed with distilled water to remove sericin proteins and wax. Extracted proteins were dried and dissolved in a warm 9 M LiB solution and kept for 2 h. The silk solution was dialyzed using distilled water for 2 days using a dialysis membrane with a molecular weight cut-off of 3,500 (Thermo Fisher Scientific, Waltham, MA, United States). The ilk solution was then concentrated. To produce the hydrogels, an aliquot of the concentrated silk solution was introduced into a silicon cylinder and the cylinder was immersed in methanol for 12 h at room temperature (25–27°C). The resulting silk hydrogel was mixed with 1 µM of the different molecular beacons used in this study.

### Printing Silk-Based Tridimensional Microarrays

The silk hydrogel containing the different molecular beacons described in the previous sections was utilized to manually print the microarrays using a Gilson P2 micropipette and the TIRF microarrayer shown below in panel A (TIRF Labs, Cary NC, United States). Typically, each microarray spot was approximately 1 mm in diameter and about 1 mm in height when dispensing 1 µl on each spot (phase contrast image from the microscope in panel B).

### Reading and Analyzing the Microarrays

The microarray slides were mounted on a conventional epifluorescence inverted microscope (Nikon Instruments, Japan) using a ×10 objective. Images were acquired using an inexpensive 5 MP CCD camera from Amscope (MT5000-CCD). All microarray data were analyzed using the ImageJ line profile tool and Igor Pro v7 (WaveMetrics, Portland, OR, United States). A target was applied to the microarray slide using a Gilson micropipette.

### Shallow Angle Illumination

We use an illumination device that excites the sample from a lateral pathway (TIRFLabs, Cary, NC). The device can be mounted on a conventional epifluorescence inverted microscope (Asanov et al., 2012). The use of the shallow angle illumination device results in having independent excitation and emission optical pathways, promoting reduced stray light and cross talk between the excitation and emission channels (Asanov et al., 2012). Shallow angle illumination results in the excitation of the sample only a few microns above the surface of the coverslip, preventing the excitation of the bulk solution. With each microarray spot having a height of 1 mm, with shallow angle illumination, we ensure that the excitation light is focused primarily on the microarray and not the solution above. A detailed description of the optical properties and physics behind shallow angle illumination can be found elsewhere (Asanov et al., 2012; Asanov et al., 2010).

### Characterization of the Tridimensional Geometry of Silk Hydrogel Droplets

Silk hydrogel was prepared using a synthetic molecular beacon for H1 (MB_H1_) in which the 5′ quencher was not included in order to have a MB that emits fluorescence continuously in the absence of the target. Silk hydrogel microarrays were printed with this MB and confocal microscopy experiments were conducted to reconstruct the tridimensional shape of the droplets. An Olympus FV1000 confocal microscope was utilized for the morphological characterization of the hydrogel droplets. Images of the entire microarray were obtained with a low magnification (×20) objective and a resolution of 2,048 × 2,048 pixels in the x-y plane. The z-plane optical slices were obtained every 200 nm. The entire image stack was loaded into Imaris v8.1 (Oxford Instruments, United Kingdom) for the tridimensional reconstruction and plane projections illustrated in Supplementary Figure S1.

## Results

### Designing Molecular Beacons to Hybridize at Room Temperature

We use our shallow angle illumination device mounted on a conventional epifluorescence inverted microscope (material and methods) (Asanov et al., 2012). Unlike conventional microarray readers, which are basically confocal microscopes, our device utilizes a camera to acquire the fluorescence of the entire microarray in one step (conventional microarray readers read one pixel at a time). This procedure provides a time course of fluorescence increments, that reflects the interaction between the bio analyte (target) and the molecular beacon (MB).

**FIGURE 1:**
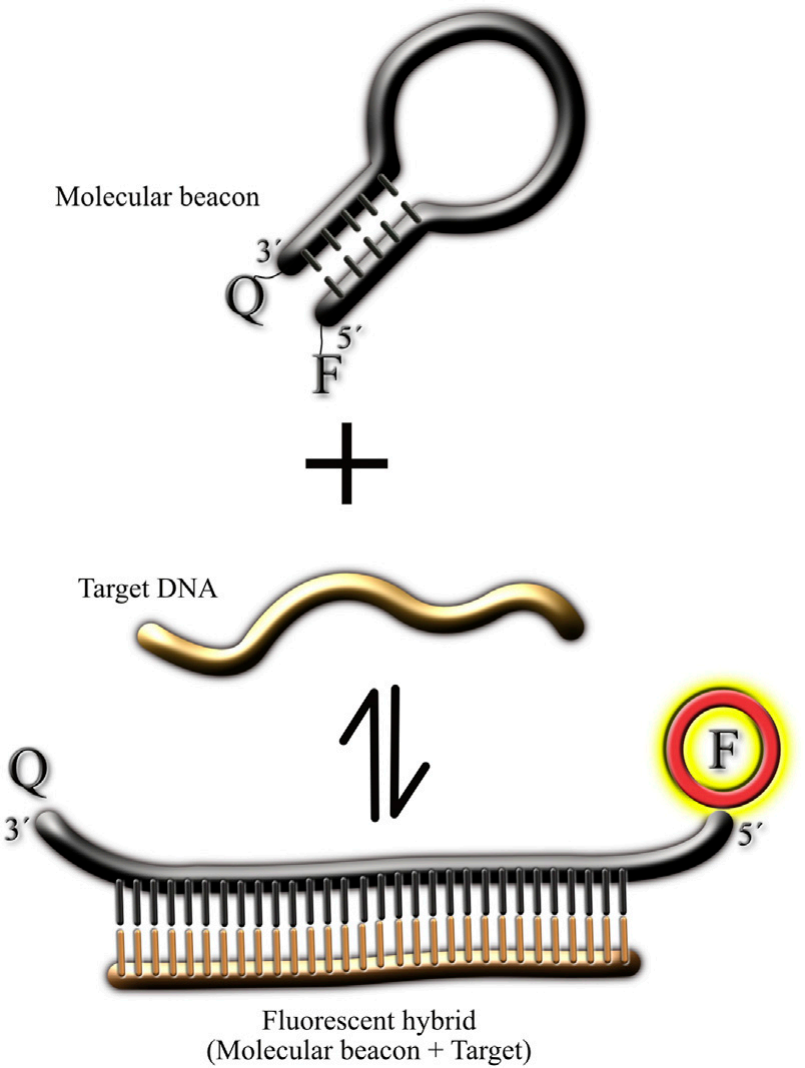
Cartoon illustrating the different molecular conformations of molecular beacons. The figure illustrates the molecular beacon in the non-fluorescent state (hairpin) in the absence of the target. Upon target addition, the 3′ and 5′ ends of the molecular beacon separate from each other, resulting in light emission by the fluorophore. In all the MB synthesized for this study the fluorophore was at the 5′ end and the quencher at the 3′ end (Material and Methods).

Figure 1 illustrates the typical reaction of a MB and its target. Under resting conditions (without the presence of the target), the MB should be in the rest (non-fluorescent) state, forming a hairpin which brings together the fluorophore and its quencher, preventing light emission in this way. Upon the addition of the target, the bimolecular association MB-target is formed, resulting in the spatial separation of the fluorophore and its quencher, and therefore light emission. Ideally, the bimolecular MB-target state should be more favorable to prevent dissociation of the duo, resulting in reduced fluorescence signal. To ensure that the bimolecular state is favorable in this reaction, the stem from the MB must be carefully designed. Typically, a high GC content in the stem will prevent spontaneous separation of the fluorophore and its quencher but will also make more difficult the formation of the MB-target binomial (since the thermodynamics would favor the hairpin state). Since traditionally MBs have been used in PCR reaction, where temperature is controlled by a thermal cycler, the MBs used in PCR reactions have typically high GC content in their stems (70%–100%). This high GC content would not work in assays conducted at room temperature, and therefore is not suitable for point-of-care diagnostics using simple equipment. In fact, the thermal transition of MBs is such that at room temperature, there is basically no florescence emitted by the probe (Tyagi and Kramer, 1996).

To overcome this design, we explored the GC content usable for MB-target associations at room temperature (25–27°C). We also explored several stem lengths to identify the best combination of stem length and GC content to favor MB-target association at room temperature.

The MBs contained in our silk hydrogel were printed on the surface of silica microarray glass (1 mm thick) and the slide was mounted on the inverted microscope and fluorescence was monitored using a CCD camera (Material and Methods) and our shallow angle illumination device. Silk printed hydrogels form a tridimensional droplet of approximately 1 mm in diameter and 700–800 microns in height (Supplementary Figure S1). A typical fluorescence experiment is illustrated in Supplementary Video S1. The mean fluorescence intensity was plotted over time to illustrate the time course of association of the MB-target and its kinetics. The video shows how the area of each microarray spot was measured using a region of interest (ROI) in ImageJ (material and methods). For each microarray experiment a replica of at least six spots was measured to obtain the mean ± standard deviation. At least 5 independent microarrays were evaluated for each of the conditions explored. Supplementary Video S2 shows the time course of fluorescence increments upon target application.

Because leakage of the MBs from the hydrogel would result in reduction in the fluorescence intensity of the microarray, we characterized the time course of MBs leakage from the hydrogel. Supplementary Figure S2 (and Supplementary Video S3) shows that the leakage of the MBs is a very slow process that does not interfere with the fast association times of MB-target interactions.

### Effect of GC Content in the Stem From the MB in the Association Constants MB-Target

To explore the effect of the GC content in the stem domain of MBs, we designed 3 MBs with different stem GC content. In all three cases, the stem length was 5 nucleotides long, and we varied the GC content from 40, 60, and 100%.

**FIGURE 2:**
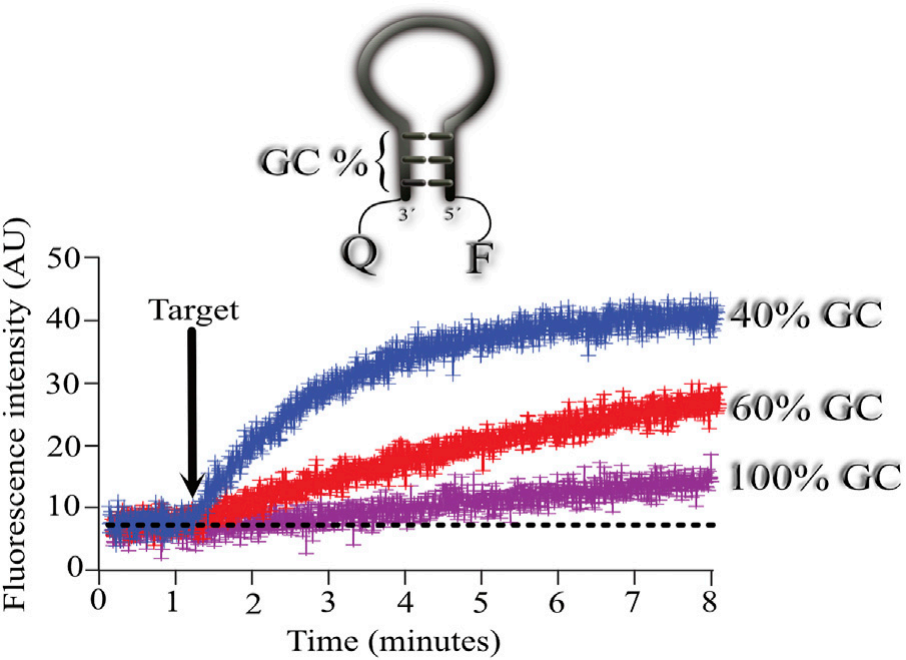
Effect of the percentage of GC content on the hybridization kinetics of the MB-target. Mean ± standard deviation from 6 independent microarrays measuring the mean fluorescence from at least 6 spots for each microarray. Three MBs with different stems were assayed consisting of 40 (blue), 60 (red) and 100 (fuchsia) % GC content in their stem. Arrow indicates the time of target application to the microarrays. Mean fluorescence was obtained from regions of interest (ROI) using the plot profile function from ImageJ for each microarray spot and measured overtime (see Supplementary Video S1). Then, means from all spots measured overtime were used to calculate the final mean ± standard deviation (see Supplementary Video S1).

As illustrated in Figure 2, using a stem with 100% GC resulted in very slow association kinetics between the MB and its target (shown in fuchsia). Using the same target, we next explored the association between the MB with 60% GC content and its target (shown in red). In this case, the association kinetics were faster, with the fastest association obtained when using 40% GC in the stem (shown in blue).

### Effect of the Length of the Stem From the MB in the Association Constants MB-Target

**FIGURE 3:**
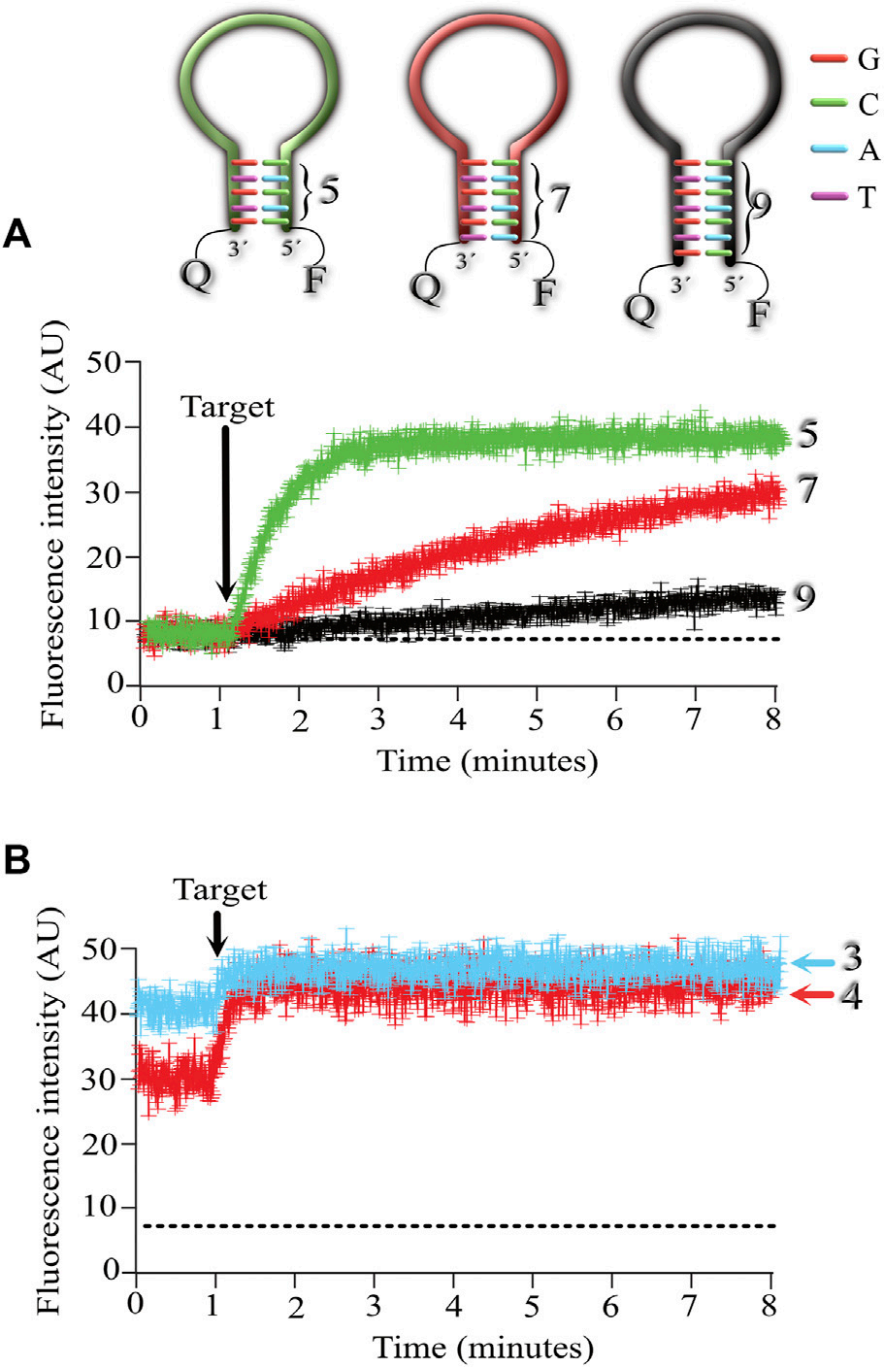
Exploring the effect of the stem length on the association kinetics of the MB-target. Five MBs were synthesized with stem lengths of 3,4,5,7, and 9 nucleotides long. Fluorescence kinetics (Mean ± standard deviation) was measured from 6 independent microarrays, measuring the mean fluorescence from at least 6 spots for each microarray. **(A)**, kinetics of MBs with stem lengths of 5 (green), 7 (red), and 9 (black) nucleotides. **(B)**, kinetics of MBs with stem lengths of 3 and 4 nucleotides. Notice that using 3 (light blue) and 4 (red) nucleotides in the stem results in higher background fluorescence (baseline level). Arrow indicates the time of target application to the microarrays.

In the next series of experiments, we were interested in exploring if the length of the stem affected the association kinetics between MB and its target. For this purpose, we designed 3 MBs with stems 5, 7 and 9 nucleotides long (Figure 3A). Using a stem of 9 nucleotides (even when the stem contained 40% GC as in the previous experiments), the association kinetics were very slow (shown in black). Reducing the stem length to 7 nucleotides sped up the association kinetics between the MB and its target (shown in red), but the fastest association was obtained with MBs formed by a stem 5 nucleotides long (shown in green).

Reducing the length of the stem to 4 nucleotides resulted in increased background fluorescence in the absence of target, indicating that a portion of the MB was in the open (fluorescent) conformation (Figure 3B). A greater background fluorescent level was observed when using MBs with 3 nucleotide long stems (Figure 3B).

These results indicate that the fastest association kinetics at room temperature are obtained with MBs formed by a stem 5 nucleotides long and with a GC content of 40%. This MB configuration did not compromise the MB conformation in resting (non-florescent) conditions, which results in low background levels and reduced signal-to-noise ratios in the absence of the target.

### Effect of the Overlap From the Target in the Stem From the MB in the Association Constants MB-Target

After identifying the best configuration for MBs consisting of 5 nucleotide long stems with 40% GC content, we proceeded to determine the effect of overlapping the target only in the loop region of the MB versus overlapping the loop and invading a portion of the stem.

**FIGURE 4:**
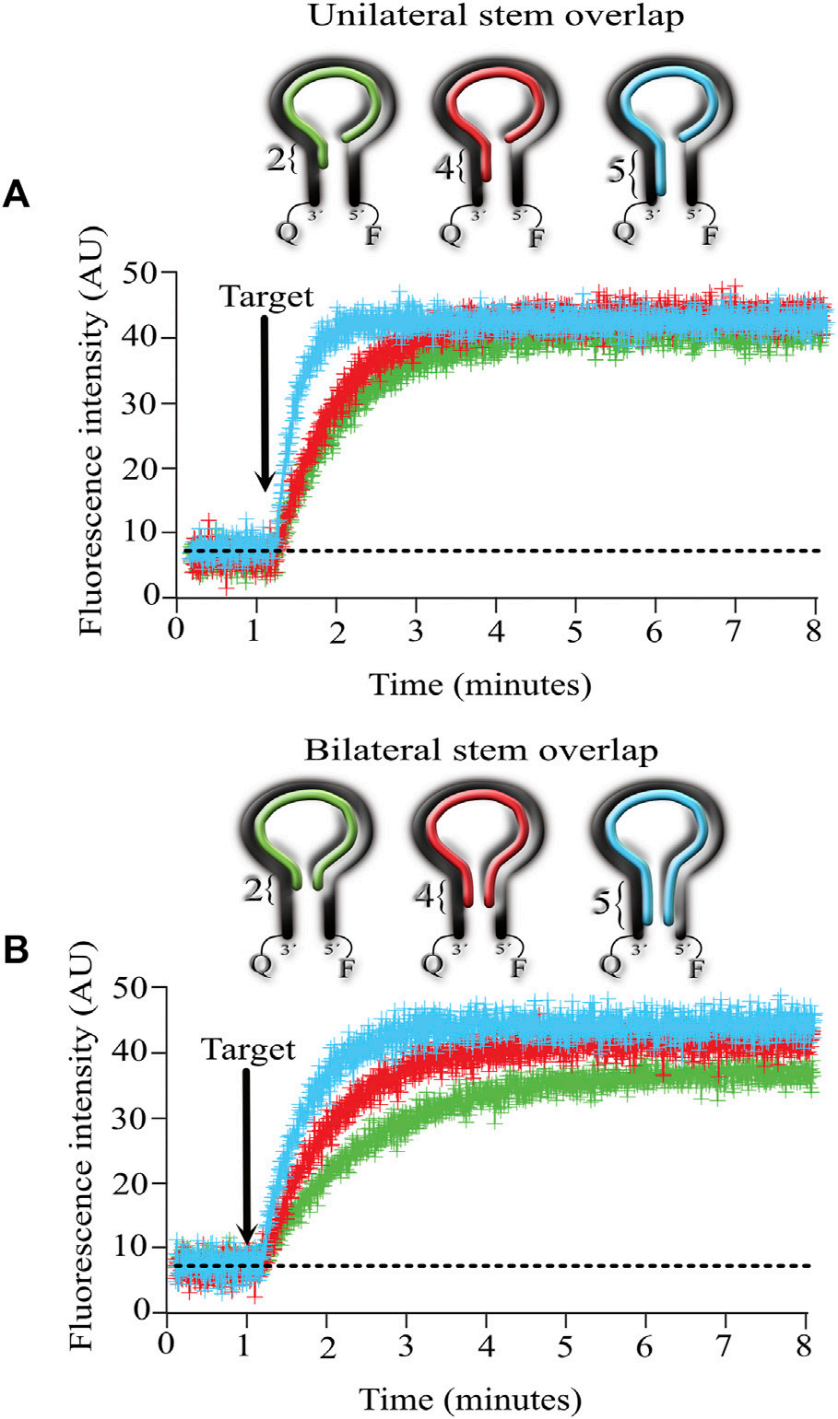
Effect of overlapping the stem with the target complementary sequence on the kinetics of MB-targe association. **(A)**, target overlapping 2 (green), 4 (red) and 5 (light blue) nucleotides of the stem from the MB in the 3′ direction (unilateral). **(B)**, target overlapping 2 (green), 4 (red) and 5 (light blue) nucleotides of the stem from the MB in the 3′ and 5′ directions (bilateral). Values show the mean ± standard deviation from 6 independent microarrays, measuring the mean fluorescence from at least 6 spots for each microarray.

To conduct these experiments, we designed targets that hybridize with the entire loop and with 2, 4, and 5 nucleotides from the stem on one side (unilateral, Figure 4A) or with the same procedure but overlapping on both sides of the stem (bilateral, Figure 4B).

The results obtained indicate that overlapping unilaterally or bilaterally made no difference in the association kinetics between the MB and its target. However, the number of nucleotides invading the stem did make a difference, with increasingly faster kinetics as the number of invading nucleotides augmented. Unilaterally or bilaterally, the fastest kinetics were obtained when the target complemented the entire stem sequence (5 nucleotides long, compare the blue lines in 4A and 4B).

The higher background fluorescence reported here agrees with previously published (Tsourkas et al., 2003) results indicating that MBs with stems consisting of 4 nucleotides still recognize their target but show high background fluorescence in the absence of the target.

### Identifying Influenza Virus Subtypes Using MBs and Silk Microarrays

To explore the feasibility of using our MB-silk microarray technology in molecular diagnostics, we designed experiments directed at identifying cDNA sequences from different influenza virus subtypes. To design the most selective MBs for influenza we conducted in silico analysis of sequences to identify unique signatures that may serve in the design of MBs selective for each influenza virus subtype (material and methods). After all the in-silico analysis, we identified specific sequences for hemagglutinin (H1, H2 and H3) and neuraminidase (N1, N2) genes from human and two genes from swine origin (H1s and N1s). The sequences of these MBs are shown in the Material and Methods section.

**FIGURE 5:**
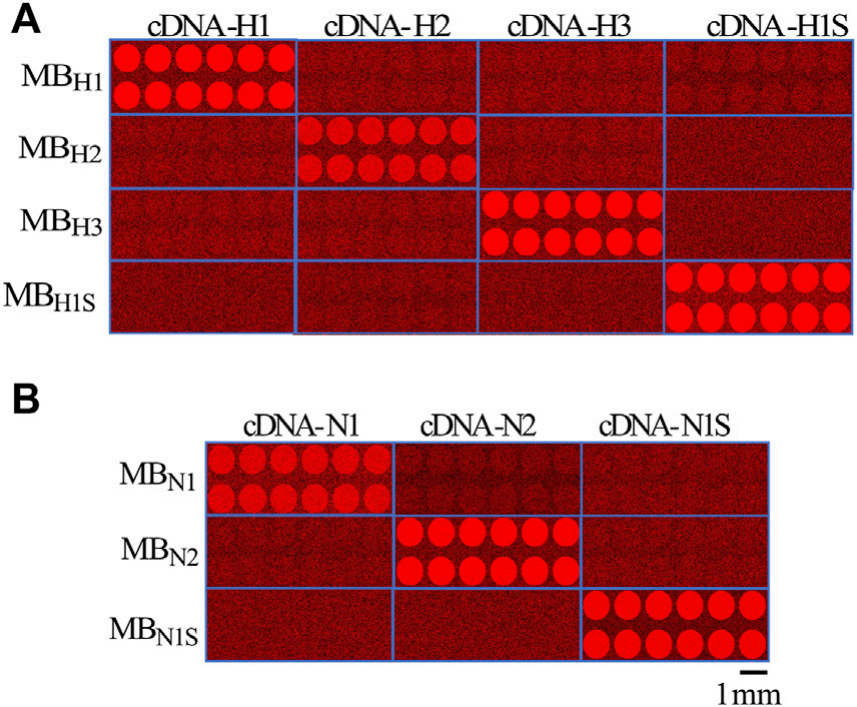
Images from microarrays illustrating the selectivity of the MBs to identify their respective targets. **(A)**, images obtained from the microarrays illustrate the selectivity of the MBs to identify their specific target cDNA for hemagglutinin H1, H2, and H3 from human and swine H1s (each sample was printed 12 times to ensure reproducibility of the array. Microarrays were acquired separately for each gene and then combined into a single image as shown in A. **(B)**, similar procedure to the previous study in A, but in this case using the cDNA and MBs for the neuraminidase N1 and N2 from human and swine N1s. All microarray images were color mapped to red to enhance the signal to the naked eye. The spatial scale shows 1 mm.

Figure 5A illustrates the fluorescence changes induced after target administration for the different hemagglutinin (H1, H2, H3 and H1s) cDNAs, and Figure 5B shows the results obtained for the neuraminidase (N1, N2 and N1s).

As illustrated in the figure, only the MBs designed to interact with their respective targets fluoresced upon target application. No cross-reactivity was observed between the hemagglutinin or neuraminidase MBs; they responded only to their respective targets. To demonstrate the reproducibility of the response, we printed 12 spots for each MB (Figure 5). The time course of the fluorescence change in the MBs is more clearly illustrated in Supplementary Video S4. The video also shows the selectivity of the MBs for their specific target. When the cDNA for N1 is applied, the MBs do not fluoresce since they are designed for N2. Only when N2 is applied the respective MB emit fluorescence (approximately halfway through the video sequence).

### Sensitivity and Selectivity of the MBs in the Identification of Influenza Virus Subtypes

**FIGURE 6:**
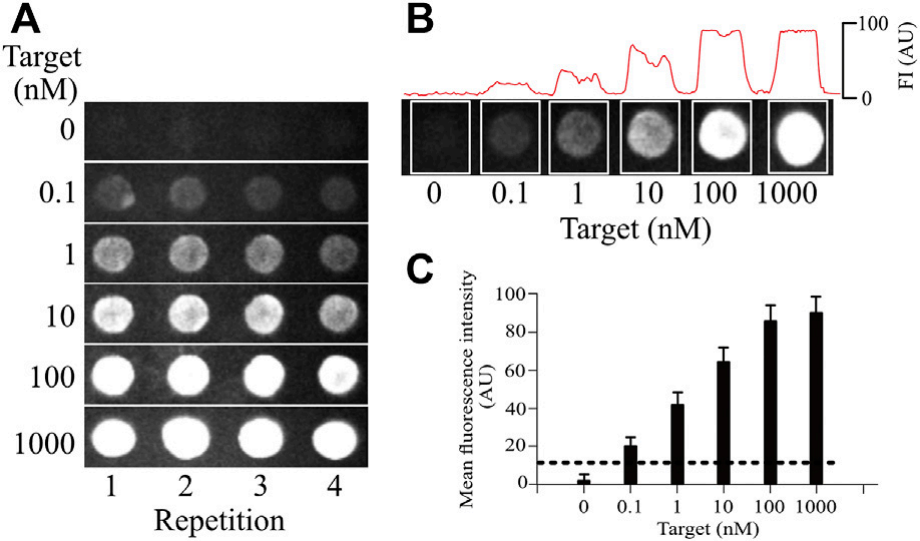
Sensitivity of the silk-based hydrogel microarrays. **(A)**, testing different concentrations of the target to explore the sensitivity of the MB (0.1, 1, 10, 100, 1,000 nM). Each microarray row (replica) is independent. All concentrations were tested on independent microarrays (not subsequently added). **(B)**, measuring the fluorescence on each microarray spot to obtain the mean value, which was used to determine the sensitivity limit. All concentrations were tested on independent microarrays (separated by the white boxes). **(C)**, the sensitivity limit obtained from the measurements as shown in B. Each bar shows the mean ± standard deviation from at least 5 spots in the same microarray and from 6 independent microarray replicas. The dotted line shows the threshold for noise, determined as 3 standard deviations from the background signal. Above, this dotted line signal is considered different from background noise (illustrated in the 0-target concentration in A and B).

To determine the sensitivity of our microarray method, we conducted experiments aimed at identifying the smallest amount of sample that our MBs can detect (Figure 6).

Maintaining a constant concentration of 1 µM MB in the tridimensional silk microarray, we varied the concentration of the cDNA for the different genes of influenza. As illustrated in Figure 6, the minimum amount of cDNA detected under these conditions was 0.1 nM (100 p.m.). The dotted line shows 3 times the standard deviation from noise as a threshold to separate positive signals from the background. Similar values were obtained for all influenza genes explored, indicating that the sensitivity of the method was not dependent on the target to be identified but rather a property of the MB concentration and the optical properties of our silk microarray. The sensitivity of our system is higher than what has been reported for conventional microarray technology, with our microarray technology showing at least 5 times greater sensitivity (Thissen et al., 2014).

## Discussion

Even though many molecular tools are used daily in laboratories for research purposes, only a handful have made their way into the diagnostics area. There are many reasons why most molecular tools have not made it to clinical practice (North et al., 2010). Some of the reasons include: the complexity of the equipment used with these molecular tools, the need for a specialized technician to operate such equipment, the difficulty of analyzing the results obtained and transforming them into a diagnostic, the lack of portability of the equipment, the difficulty of standardizing the method for diagnostic procedures, the complexity of sample preparation, the need for expensive reagents, etc. (Giljohann and Mirkin, 2009).

Molecular beacons (MB) have been extensively used in research but also in molecular diagnostics in combination with real-time PCR (Abravaya et al., 2003; Broude, 2002; Fang et al., 2000; Kim et al., 2008). In fact, real-time PCR has become the gold standard for diagnostics of infectious diseases (the best example is the current coronavirus pandemic) (Floriano et al., 2020).

Some studies have reported the use of MB in reagentless experimental diagnostics (Beaucage, 2001; Brown et al., 2000). In these studies, the MBs are immobilized directly onto the glass surface of the microarray slide. Unfortunately, this procedure results in reduced fluorescence and high background levels (Teng and Libera, 2018). To prevent the quenching of the MBs by the glass surface, recently a novel method has been implemented in which the MBs are immobilized in microgels produced from polyethylene glycol (PEG) (Teng and Libera, 2018). These microgels not only localize the probes to specific surface positions but also maintain them in a waterlike environment (Teng and Libera, 2018). The microspheres used also exhibit a lensing behavior, resulting in the enhancement of the fluorescence emitted by the MBs.

In the present study, the 3D printed silk hydrogel microarray spots produce a similar lensing behavior. A geometrical lensing effect occurs in curved surfaces arising from the nature of parallel lines in spherical and hyperbolic space (Schönhöfer and Glotzer, 2022). This effect is further enhanced by the fact that the refractive index of water and silk are almost identical, and thus the entire hydrogel behaves as a homogenous material.

In the present study, we report a novel method to produce MB-based microarrays using silk hydrogel for encapsulation. This method also produces a friendly biomolecular waterlike environment. Because silk’s refractive index is similar to that of water (1.3), we have observed a strong lensing behavior with light propagating properties which enhances the fluorescence emitted by the MBs upon target hybridization. The refractive index of PEG is slightly higher (1.4) than that of water (1.33) or silk (1.34). The similar refractive indexes of silk and PEG may explain the microlens effect and fluorescence enhancement observed in our study.

The use of shallow angle illumination reduces the excitation of the bulk solution and separates the excitation and emission pathways, reducing stray light and channel crossed talk, as we have previously reported (Asanov et al., 2012).

By studying the properties (length and GC content) of the stem of MBs, we were able to identify the best combination that favors association of the MB-target at room temperature. Our studies indicate that 5 nucleotides long is the minimum length to prevent background fluorescence from the MB in the absence of a target. 40% GC content in the stem is critical to accelerate the association kinetics of the MB-target complex at room temperature. Higher GC content resulted in slower association kinetics, which is not desirable for rapid point-of-care diagnostics.

This configuration of the stem length and GC content in the MB did not alter its selectivity for the specific target, as we have shown here.

Even though our microarray technology is not as sensitive as that from realtime PCR, the use of silk hydrogel in tridimensional microarrays provided higher sensitivity than previously reported obtained with conventional microarrays (Thissen et al., 2014).

One of the main disadvantage of using hydrogels is that they cannot be stored for long periods of time because they tend to dehydrate and shrink. We have observed hydrogel dehydration and shrinkage within the first 48 h of storage at room temperature (once printed on the coverslip). Hydrogel dehydration can be prevented by keeping the microarray slides at 4°C until used.

In previous studies using MBs in hydrogels, the MBs have been immobilized in the hydrogel by chemical reactions to prevent diffusion (Teng and Libera, 2018). We have used the MBs without chemical immobilization and found that MB leakage outside the hydrogel is negligible as long as the printed microarray is maintained in a humidity-controlled environment but not immersed in water (Supplementary Figure S2 and Supplementary Video S3). Target application in a water solution facilitates MB diffusion outside the hydrogel, but this phenomenon is very slow and takes several hours while the signal produced by the association MB-target takes place within minutes of target application (Supplementary Figure S2 and Supplementary Video S3).

Although in the present study we used a conventional fluorescence microscope coupled to our shallow angle illumination device to capture the fluorescence emitted by the MBs in the microarray, simpler portable detectors can be easily implemented using inexpensive cMOS cameras and LEDs in combination with adequate excitation and emission filters.

## Data Availability

The datasets presented in this study can be found in online repositories. The names of the repository/repositories and accession number(s) can be found at: http://www.ncbi.nlm.nih.gov/genomes/FLU/FLU.html.

## Supplementary Material

Supplementary Figure S1 Tridimensional characterization of the geometry of silk hydrogel droplets. The molecular beacon for H1 (MB_H1_) was synthesized without the 5′ end quencher (Material and Methods), to have a MB that emits fluorescence continuously (without the target present). Silk was prepared with this MB and droplets were printed using the microarrayer (Material and Methods). Confocal microscopy studies were conducted with the microarrays printed with this MB. Optical slices were obtained every 200 nm, and 3D images of the microarray spots (droplets) were rendered using Imaris 8.1 (Oxford Instruments). The height of the droplets is color coded to indicate the different heights of each microarray spot. Panel A shows the droplets visualized from one side. Panel B after rotating the horizontal plane 90 degrees and panel C after rotating the vertical panel 90 degrees to obtain a top view. The scale in C shows 1 millimeter (1 mm).

Supplementary Figure S2 Leakage of the MBs from the hydrogel is a very slow process. **(A)**, examples of microarrays printed with the silk hydrogel prepared with the molecular beacon for H1 (MB_H1_) synthesized without the 5′end quencher (Material and Methods). Notice the very small reduction in fluorescence over time that after 20 minutes amounts to a reduction in fluorescence intensity of less than 1% of the initial fluorescence values (see also Supplementary Video S3). **(B)** Mean ± standard deviations of the fluorescence intensity obtained from at least 8 microarray spots from 4 independent experiments. In red are shown the values for the MB leakage experiment and in green the values for a typical MB_H1_/cDNA-H1 reaction (shown inside the gray rectangle). Notice that the leakage of the MB_H1_ is negligible during this 7-minute time window. The black dotted line shows the baseline fluorescence, and the blue dotted line shows the initial mean fluorescence value of the MB_H1_ without the quencher to highlight more clearly the amount of MB leakage during the time course of the experiment (24 min).

Supplementary Video S1 Video illustrating the use of the procedure plot profile from ImageJ, utilized to calculate the mean fluorescence for each microarray spot. All spots were measured individually and later compounded into the mean ± standard deviation from 6 independent microarray replicas.

Supplementary Video S2 Video illustrating the reproducibility of the microarray using multiple replicas of the same MB. The spatial scale (white line) shows 1 mm, and the time scale is in minutes. A red arrow indicates the time of target application. Notice that all 9 replicas respond to the target application with similar time courses.

Supplementary Video S3 Time course of the leakage of MB from the silk hydrogel. A molecular beacon synthesized without the quencher was used to illustrate MB leakage from the hydrogel. Since the quencher is not present, the MB emits florescence continuously. Notice that after 25 minutes, the fluorescence inside the hydrogel remains almost intact, reflecting limited leakage of the MB. Notice that most reactions to MB-target conclude within 9 minutes after target application (video 2).

Supplementary Video S4 The video shows 3 replicas for each of the two MBs printed, one designed to detect hemagglutinin (H1) and neuraminidase (N2). Notice that upon application of the cDNA for H1 (cDNA-H1), only the replicas containing the MB (H1) respond to the stimulus. Later in the video, the cDNA for N1 (cDNA-N1) is applied, but since the microarray contained no MB for this cDNA, no response was obtained. At the end of the video, the cDNA for N2 (cDNA-N2) is applied, to the microarray and the replicas containing the MB (N2) printed response to this application. This video illustrates the selectivity of the MBs designed for each specific target.
