# Supplementary figures and images for: Design of Hydrogel Silk-Based Microarrays and Molecular Beacons for Reagentless Point-of-Care Diagnostics
Source: Front Bioeng Biotechnol. 2022 Jul 22;10:881679. doi: 10.3389/fbioe.2022.881679 (PMC9361048; doi:10.3389/fbioe.2022.881679)

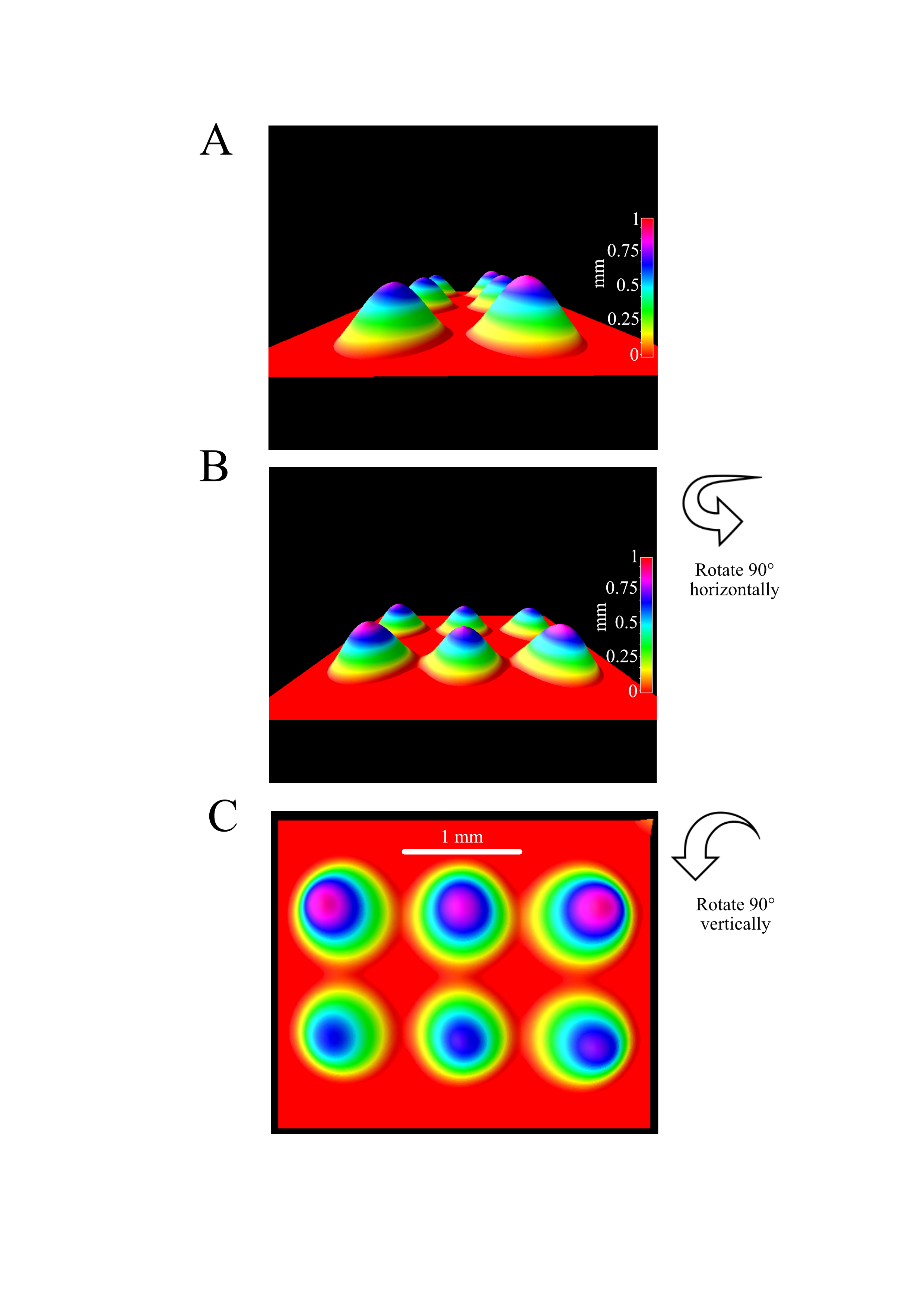

Supplement: Supplementary file 1 [file Image1.tiff]

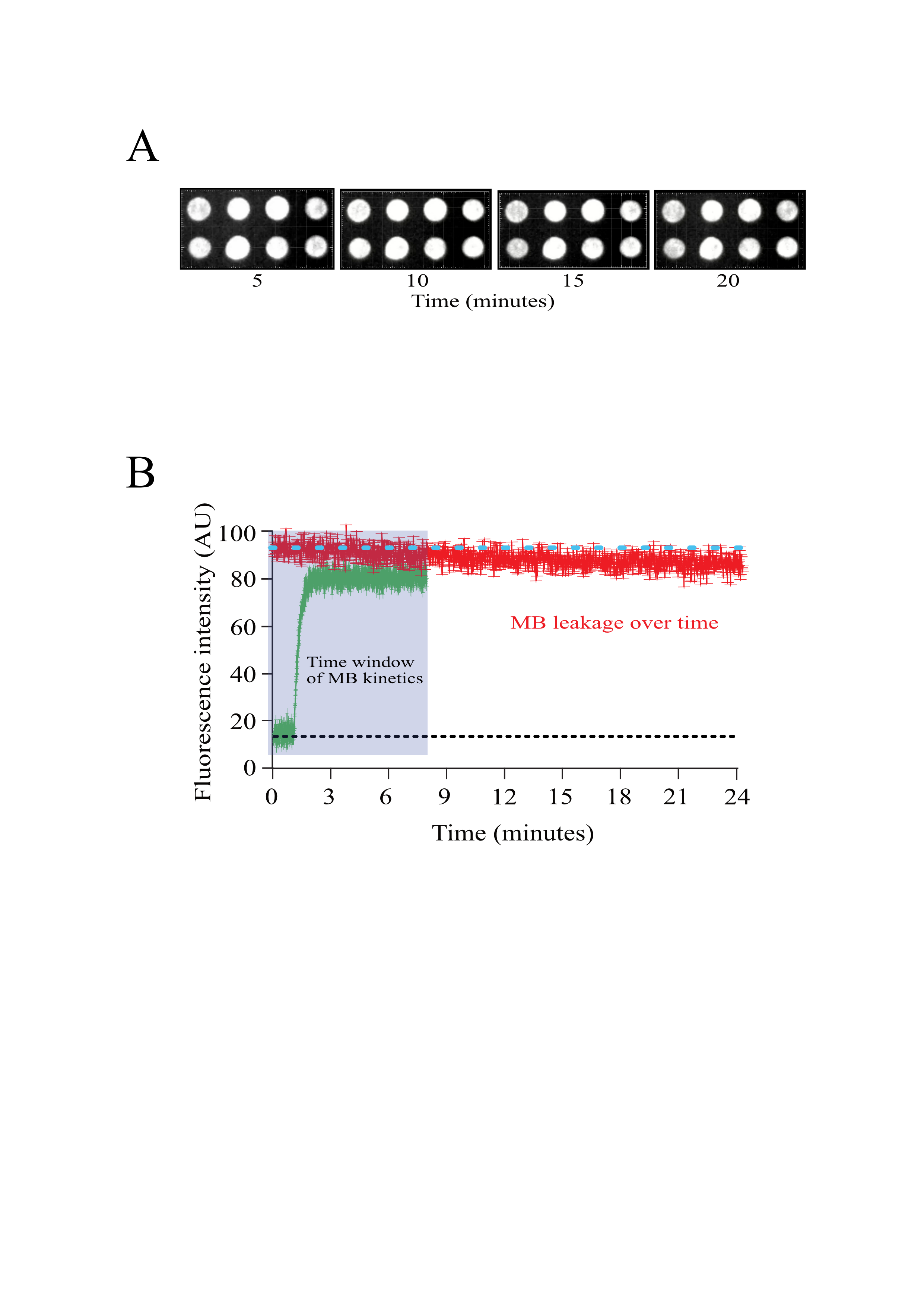

Supplement: Supplementary file 3 [file Image2.tiff]
